# Optimal ventilation strategy

**DOI:** 10.1186/1824-7288-41-S1-A19

**Published:** 2015-09-24

**Authors:** Gianluca Lista, Azzurra La Verde, Francesca Castoldi

**Affiliations:** 1 Neonatal Department - Ospedale dei Bambini “V. Buzzi”, Azienda ICP, Milano, Italy

## 

Premature delivery, is always associated to the failure of respiratory transition and a delayed achievement of an adequate functional residual capacity. For this reason preterm babies (expecially the Extremely Low for Gestational Age -ELGA- infants) frequently need a respiratory support. Non-invasive ventilation (NIV) is widespread used in the management of respiratory distress even in ELGA infants without increasing of neonatal mortality or neurological impairment.

The most recent meta-analysis and reviews on NIV approach demonstrated that NIV is a valid alternative to mechanical ventilation (MV) in the management of respiratory failure and resulted in significant reductions in the incidence of Bronchopulmonary dysplasia (BPD) amongst surviving infants [1,2]. 

Nevertheless, even if non-invasive respiratory support seems to be very efficacious in preterm babies, sometimes ELGA infants require MV.

Pressure-limited ventilation is yet the standard mode in neonatal intensive care unit, because the attempts to use traditional volume-controlled ventilation it has been often impractical in very small preterm infants. Moreover pressure-limited ventilation continues to be the primary mode of ventilation in neonates because of its relative simplicity and ability to ventilate effectively despite large ETT leak.

The major disadvantage of pressure-limited ventilation is that the tidal volume (VT) varies with changes in lung compliance (e.g after surfactant therapy). The consequences of such rapid improvements in compliance are inadvertent hyperventilation and lung injury from excessively large VT (volutrauma); inadvertent hyperventilation may induce hypocapnia, with high risk of cerebral damage. In literature there are extensive evidence that excessive volume, rather than pressure, is the key determinant of ventilator-induced lung injury (VILI). Inadequate (too small) VT also causes significant problems (atelectrauma): in particular inefficient gas exchange due to increased dead-space to VT ratio. Therefore in the last years volume-targeted ventilation has become an important standard of care in neonatal and pediatric respiratory support too.

There are many volume-targeted ventilation modes to be used in neonatal period, but Volume Guarantee (VG) ventilation, it is the most extensively studied volume-targeted ventilation mode in the neonate with respiratory distress. 

The Cochrane review on volume-targeted ventilation resulted in significant reductions in duration of ventilation, rates of pneumothorax, significant reduction in intraventricular haemorrhage (IVH) and a reduction in the incidence of BPD amongst surviving infants, of borderline statistical significance [3]. The VG ventilation plus adequate PEEP and an open lung strategy [4], seems to be an adequate alternative to High frequency Oscillatory Ventilation (HFOV) in mechanically ventilated infants for RDS.
